# Positive end-expiratory pressure and prone position alter the capacity of force generation from diaphragm in acute respiratory distress syndrome: an animal experiment

**DOI:** 10.1186/s12871-022-01921-0

**Published:** 2022-12-02

**Authors:** Andi Muhammad Fadlillah Firstiogusran, Takeshi Yoshida, Haruka Hashimoto, Hirofumi Iwata, Yuji Fujino

**Affiliations:** 1 The Department of Anesthesiology and Intensive Care Medicine, Osaka University Graduate School of Medicine, Suita, Japan; 2 Osaka, Japan

**Keywords:** Spontaneous breathing, Prone position, PEEP, ARDS

## Abstract

**Background:**

Spontaneous breathing potentially injures lungs and diaphragm when spontaneous effort is vigorous in acute respiratory distress syndrome (ARDS) while immobility also has risks of Intensive Care Unit (ICU) acquired weakness and diaphragm atrophy. Thus, ventilatory strategy to mitigate strong spontaneous effort should be promptly established without a systemic use of neuromuscular blocking agent. Here, we investigated the impacts of positive end-expiratory pressure (PEEP) and body position on the capacity of force generation from diaphragm following bilateral phrenic nerve stimulations in a rabbit ARDS model.

**Methods:**

Using lung-injured rabbits, we measured 1) transdiaphragmatic pressure by bilateral phrenic nerve stimulation and 2) end-expiratory lung volume using computed tomography, under two different levels of PEEP (high, low) and body positions (supine, prone).

**Results:**

Overall, transdiaphragmatic pressure was the highest at low PEEP in supine position and the lowest at high PEEP in prone position. Compared to values in low PEEP + supine, transdiaphragmatic pressure was significantly reduced by either prone alone (the same PEEP) or increasing PEEP alone (the same position) or both combinations. End-expiratory lung volume was significantly increased with increasing PEEP in both positions, but it was not altered by body position.

**Interpretation:**

The capacity of force generation from diaphragm was modulated by PEEP and body position during mechanical ventilation in ARDS. Higher PEEP or prone position per se or both was effective to decrease the force generation from diaphragm.

## Introduction

Spontaneous breathing potentially injures lungs and diaphragm when spontaneous effort is vigorous in acute respiratory distress syndrome (ARDS) [1–5]. The key mechanism whereby spontaneous breathing may injure lungs and (potentially) diaphragm is the large excursion of diaphragm (*e.g.*, large tidal volume, high transpulmonary pressure, large pendelluft, high transvascular pressure) [6]. Silence of respiratory muscles by the systemic use of neuromuscular blockade improved survival in severe ARDS[7], but it may have a risk of ICU-acquired weakness and diaphragm atrophy[2]. Thus, a promising strategy to maintain modest level of spontaneous breathing effort (without paralyzing patients) should be established promptly [8].

Several factors increase the strength -and injury potential- of spontaneous breathing effort, including respiratory drive from cortical stimuli, chemoreceptive stimuli, and mechanical stimuli, as well as capacity of force generation from diaphragm [8–10]. Previous studies reported that higher positive end-expiratory pressure (PEEP) or prone position decreased spontaneous breathing effort in ARDS[1, 11–13], but distinct mechanism has been still unidentified, *e.g.*, decreased respiratory drive or altered capacity of force generation from diaphragm. PEEP and prone position are known to have similar effects of restoring lung volume and thus potentially may change the force–length relationship of the diaphragm, which leads to decrease spontaneous breathing effort, independent of respiratory drive.

Therefore, we investigated the impacts of PEEP and body position on the capacity of force generation from diaphragm following bilateral phrenic nerve stimulations in a rabbit ARDS model.

## Methods

The study was approved by the Laboratory Investigation Committee, Osaka University Medical School (#03,033,000). Animals were cared for in accordance with the University’s standards for care and use of laboratory animals, and all procedures were performed in accordance with the National Institutes of Health ‘Guidelines for the Care and Use of Laboratory Animals’. This study was carried out in compliance with the ARRIVE guidelines.

**Fig. 1:**
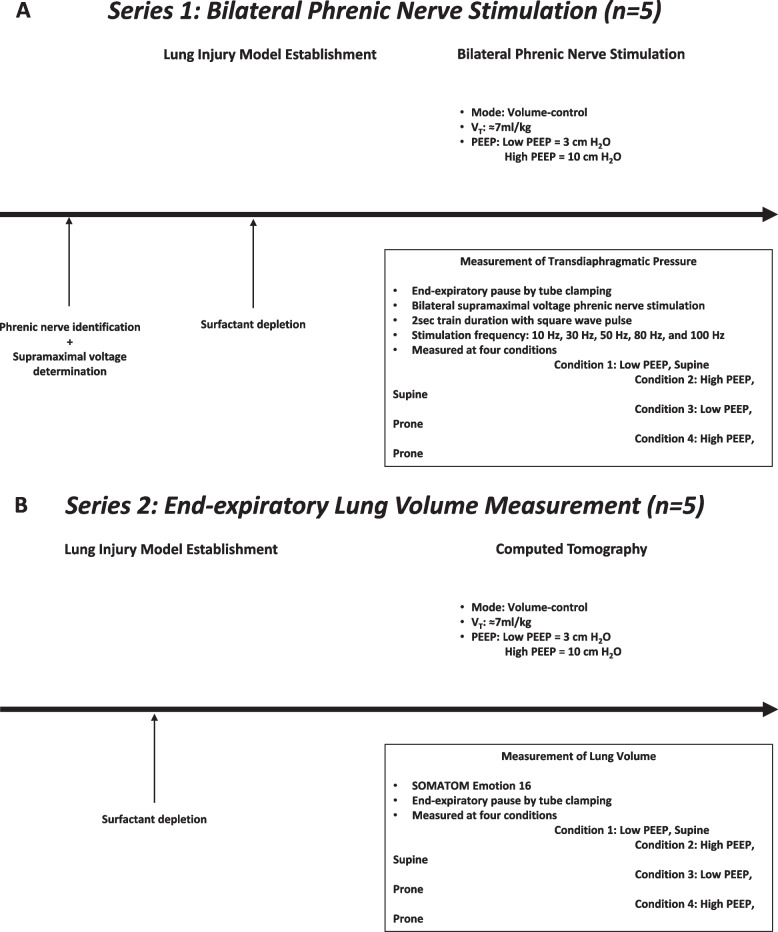
Schematic of study protocol. **A** Bilateral Phrenic Stimulation Protocol. **B** End-Expiratory Lung Volume Protocol

The schematic of study protocol is presented in Fig. 1. Ten New Zealand white rabbits (adult, male, 3.6 ± 0.2 kg; five in each series) were anesthetized with ketamine (20–30 mg.kg^−1^.h^−1^) and dexmedetomidine (2 μg.kg^−1^.h^−1^) and tracheostomized. Then, animals were ventilated in supine position with tidal volume (V_T_) 8 ~ 10 mL kg^−1^; respiratory rate 30–50 breaths.min^−1^ (targeted for PaCO_2_ ≈40 mmHg); and PEEP 3cmH_2_O, using Servo 300 ventilator (Siemens-Elema AB, Solna, Sweden) or with Puritan Bennett™ 840 (Covidien, Mansfield, MA, USA), either of which is available. An esophageal and gastric balloon (CareFusion, San Diego, USA) was inserted to estimate transdiaphragmatic pressure (Pdi), filled with air (0.3 mL as minimal non-stress volume for esophageal balloon; 2.0 mL for gastric balloon) and its position of esophageal balloon was verified as above[14].

### Lung injury

Lung injury was established by repeated saline lavage (targeted PaO_2_/FiO_2_ ratio ≈200 mmHg) at PEEP of 3cmH_2_O. All animals were temporarily paralyzed with a bolus of rocuronium bromide during establishment of lung injury. No residual effect of neuromuscular blocking agent to affect respiratory muscles was confirmed by the same amount of maximal transdiaphragmatic pressure generated with supramaximal stimuli of bilateral phrenic nerves as observed at baseline, *i.e.*, before the induction of lung injury.

### Experimental protocol

AnimalS were randomly assigned to all of four conditions as follows:

- Low PEEP, Supine;
- High PEEP, Supine;
- Low PEEP, Prone;
- High PEEP, Prone;

Measurements in each condition were performed approximately in an hour. Lungs were fully recruited by stepwise increases of airway pressure among each sequence, and then we waited for at least 30 min before starting the measurements. The animals were ventilated with volume-controlled ventilation: targeting tidal volume of ≈7 mL kg^−1^, respiratory rate between 60 to 120 min^−1^(targeted to PaCO_2_ < 50 mmHg) and F_I_O_2_ of 1.0. In our pilot study, decremental PEEP steps from 18 to 0 cmH_2_O showed that oxygenation started to decrease below PEEP of ≈10 cmH_2_O. Based on this finding, high PEEP and low PEEP was defined as 10 and 3cmH_2_O respectively.

### Series 1- Bilateral phrenic nerve stimulation

Bilateral phrenic nerves were exposed and identified by electrical stimulation with bipolar nerve block needles (Hakko, Nagano, Japan). The intensity of voltage was gradually increased until maximal value of Pdi was obtained at end-expiration (maintained lung volume by tube clamp), by stimulating bilaterally phrenic nerves with 500 ms train duration with square-wave pulse of 0.15 ms duration at 75 Hz[15]. Supramaximal phrenic nerve stimuli were defined as × 1.25 of the voltage where maximal value of Pdi was obtained[16]. The force generation from diaphragm at end-expiration (maintained lung volume by tube clamp) was evaluated by measuring Pdi for 2 s train duration with square-wave pulse of 0.15 ms duration at frequencies of 10, 30, 50, 80 and 100 Hz during bilateral supramaximal voltage phrenic nerve stimulation. Transdiaphragmatic pressure reduction was expressed with averaging how much Pdi was reduced, taken from all stimulation frequencies in each condition, compared with low PEEP, Supine.

### Series 2- End-expiratory lung volume

Computed tomography (CT) scans were performed (SOMATOM Emotion 16; Siemens Medical Solutions, Erlangen, Germany). Unenhanced helical CT scans (matrix: 512 rows × 512 columns; slices 1.5 mm; voltage 130 kV; tube current 240 mA) were performed during an end-expiratory pause with tube clumping. The images were analyzed using Osirix MD image viewer and analysis software program (version 12.5.1; Pixmeo, Geneva, Switzerland) for segmentation and volumetric measurements. The heart and diaphragm were excluded from the regions of interest. The volume of hyperinflation, normal aeration, poor aeration, and non-aeration was calculated in end-expiratory images. We identified each lung compartment according to their densities in Hounsfield Units: non-aeration (+ 100 to -200 HU), poor aeration (-201 to -500 HU), normal aeration (-501 to -900 HU), and hyperinflation (-901 to -1000 HU)[20]. The end-expiratory lung volume was calculated based on densities expressed in Hounsfield units of -1000 to -201 (excluding non-aeration), modified from previous CT study[17].

### Definitions

Definitions of pulmonary pressures was as follows.

- Peak (*inspiratory*) transpulmonary pressure, Peak transpulmonary pressure = maximal value of [airway pressure – esophageal pressure] cmH_2_O.
- Plateau (*inspiratory*) transpulmonary pressure, Plateau transpulmonary pressure = [Plateau airway pressure – end-inspiratory esophageal pressure] cmH_2_O
- Plateau pressure, airway pressure measured during a short inspiratory hold (*i.e*. zero flow phase)
- Driving Pressure = [Plateau airway pressure – PEEP] cmH_2_O
- Compliance of the respiratory system = [V_T_/(Driving Pressure)] mL·cmH_2_O.^−1^
- Compliance of the lung = [V_T_/(Driving transpulmonary pressure)] mL·cmH_2_O.^−1^
- Transdiaphragmatic pressure = [the sum of change in esophageal pressure and change in gastric pressure] This is because the absolute value of abdominal pressure was changed by body position.

### Statistical analysis

Statistical analysis were performed using SPSS 24.0.0.0 for Windows (SPSS, Chicago, IL, USA). 1-way analysis of variance (ANOVA) with repeated measures evaluated the impacts of frequency on Pdi and 1-way ANOVA evaluated the effects of condition on Pdi, respiratory parameters, and CT measures. In the post hoc analysis, a Dunnett’s test was used to compare repeated values with the value at the start of the stimulation (*i.e.* 10 Hz), and Tukey’s pair-wise multiple comparison test was used to determine condition differences. All tests were 2-tailed, and differences were considered significant when p < 0.05.

## Results

Respiratory and hemodynamics parameters are summarized in Table 1. V_T_ was low and similar (volume-controlled ventilation: ≈7 mL/kg) in all of four conditions. PEEP (and thus expiratory transpulmonary pressure) was significantly higher in High PEEP conditions, as anticipated. PaO_2_/F_I_O_2_ was the lowest in Low PEEP, Supine (80 ± 46 mmHg) and was significantly improved by increasing PEEP or changing position or both combinations. Driving pressure, respiratory system compliance and lung compliance did not significantly differ among four conditions (Table 1).

**Table 1:** Respiratory, Hemodynamics Parameters and CT measures in the Anesthetized Rabbit

Baseline	Supine Position		Prone Position	
	Low PEEP	High PEEP	Low PEEP	High PEEP
**Peak airway pressure, cmH**_**2**_**O**				
11.6 ± 1.8	21.3 ± 4.9	29.2 ± 3.6^†^*	18.8 ± 7.4	26.7 ± 3.5*
**Plateau airway pressure, cmH**_**2**_**O**				
9.7 ± 1.4	18.2 ± 4.2	26.3 ± 3.0^†^*	15.8 ± 6.8	23.8 ± 2.2*
**Positive end-expiratory pressure, cmH**_**2**_**O**				
2.8 ± 0.5	2.9 ± 1.1	10.3 ± 0.6 ^†^*	3.3 ± 0.7	10.0 ± 0.6 ^†^*
**Driving pressure, cmH**_**2**_**O**				
6.9 ± 1.0	15.1 ± 4.2	15.9 ± 3.1	12.5 ± 6.5	13.5 ± 2.3
**Tidal volume, mL/kg**				
9.0 ± 1.1	6.8 ± 0.8	7.1 ± 1.1	6.6 ± 1.2	6.6 ± 1.2
**Respiratory rate, /minute**				
36 ± 9	78 ± 4	80 ± 14	80 ± 14	80 ± 14
**Peak transpulmonary pressure, cmH**_**2**_**O**				
3.8 ± 2.8	11.8 ± 3.6	18.2 ± 3.6^†^*	9.2 ± 7.5	14.5 ± 3.1
**Plateau transpulmonary pressure, cmH**_**2**_**O**				
2.1 ± 2.4	9.2 ± 3.3	15.6 ± 2.9^†^*	6.1 ± 6.8	11.5 ± 4.3*
**Expiratory transpulmonary pressure, cmH**_**2**_**O**				
-3.1 ± 2.4	-3.7 ± 3.5	2.3 ± 3.2^†^*	-3.4 ± 4.1	0.3 ± 4.4^†^*
**Gastric pressure, cmH**_**2**_**O**				
3.9 ± 0.9	3.9 ± 1.7	5.0 ± 1.5	7.3 ± 2.4	10.1 ± 4.8 ^† ¶^
**Respiratory system compliance, ml/cmH**_**2**_**O**				
4.9 ± 0.8	1.9 ± 0.6	1.7 ± 0.3	2.4 ± 0.9	1.9 ± 0.5
**Lung compliance, ml/cmH**_**2**_**O**				
6.6 ± 1.3	2.2 ± 0.7	2.1 ± 0.6	3.1 ± 1.2	2.3 ± 0.6
**pH**				
7.35 ± 0.03	7.34 ± 0.06	7.27 ± 0.15	7.20 ± 0.19	7.24 ± 0.16
**PaCO**_**2**_**, mmHg**				
41.4 ± 3.3	42.0 ± 6.4	49.0 ± 17.0	45.0 ± 13.0	45.0 ± 11.0
**PaO2/FiO2, mmHg**				
479 ± 19	80 ± 46	403 ± 207 ^†^	303 ± 193 ^†^	400 ± 190 ^†^
**Base excess, mmol/L**				
-2.4 ± 2.7	-3.4 ± 2.7	-5.2 ± 3.9	-8.3 ± 7.5	-8.0 ± 6.6
**Hyperinflation, % of total lung volume**				
	1.7 ± 2.2	5.0 ± 3.5 ^†^*	2.1 ± 3.6	5.9 ± 2.7 ^†^*
**Normal aeration, % of total lung volume**				
	38.6 ± 17.9	71.3 ± 21.6 ^†^*	44.9 ± 14.2	66.3 ± 24.8 ^†^*
**Poor aeration, % of total lung volume**				
	32.4 ± 11.7	17.1 ± 11.8	29.1 ± 4.3	18.2 ± 13.2
**Non-aeration, % of total lung volume**				
	27.3 ± 20.9	6.7 ± 8.1 ^†^*	24.0 ± 17.8	9.7 ± 13.2 ^†^*
**Systolic arterial pressure, mmHg**				
104 ± 20	102 ± 21	105 ± 16	116 ± 19	102 ± 27
**Diastolic arterial pressure, mmHg**				
87 ± 21	84 ± 17	84 ± 21	103 ± 19	87 ± 29
**Heart rate, /minute**				
235 ± 29	210 ± 22	179 ± 18	181 ± 42	162 ± 18 ^†^

Abbreviations: PEEP = positive end-expiratory pressure; CT = computed tomography

Data are presented as mean ± standard deviation

^†^p < 0.05 compared with Low PEEP, Supine; ^¶^P < 0.05 compared with High PEEP, Supine; ^*^p < 0.05 compared with Low PEEP, Prone

**Fig. 2:**
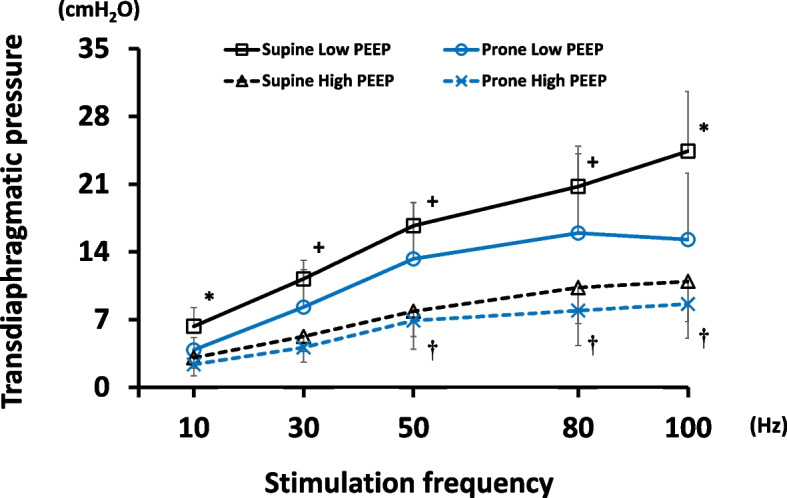
Transdiaphragmatic pressure-frequency curve in all conditions. Data represent mean ± SD. Transdiaphragmatic pressure was greater as stimulation frequency was higher in all conditions (p < 0.01 at 50, 80 and 100 Hz *vs.* 10 Hz). Overall, transdiaphragmatic pressure was the highest at low PEEP in supine position and the lowest at high PEEP in prone position. Compared to values in low PEEP + supine, transdiaphragmatic pressure was significantly reduced by either changing position to prone alone (the same PEEP) or increasing PEEP alone (the same position) or both combinations. * p < 0.05 *vs*. all conditions; + p < 0.05 *vs*. high PEEP conditions; † p < 0.05 *vs*. low PEEP + prone. Abbreviations: PEEP positive end-expiratory pressure; SD standard deviation

**Fig. 3:**
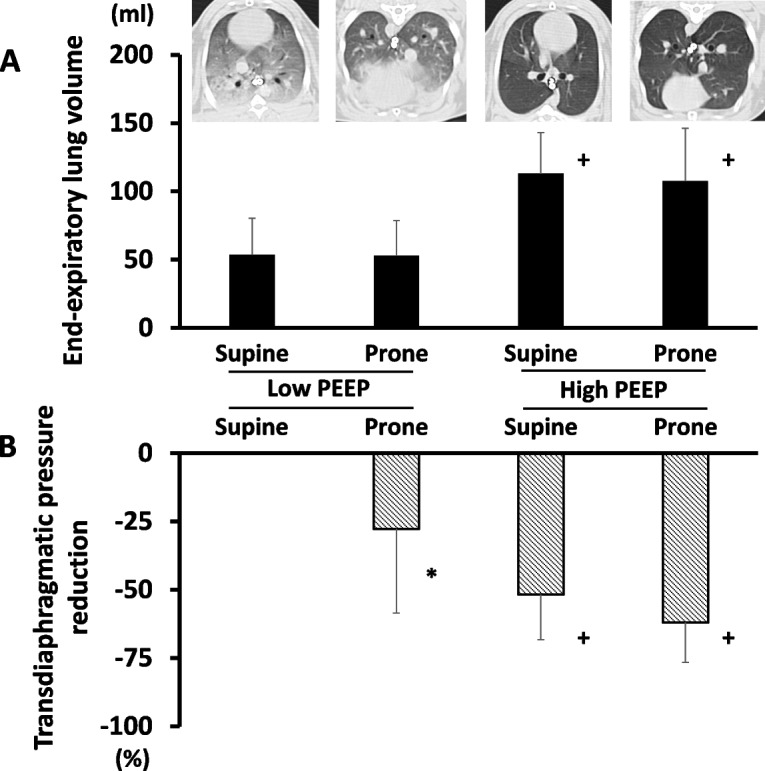
End-expiratory lung volume and transdiaphragmatic pressure reduction in all conditions. Data represent mean ± SD. End-expiratory lung volume calculated using CT was presented in all conditions (**A**). End-expiratory lung volume was higher in high PEEP conditions *vs*. low PEEP conditions. Body position did not affect end-expiratory lung volume. Representative CT images from the same subject are shown in (**A**). Representative CT images are presented. Aerated lung regions are reduced because of lung collapse predominant in dependent (dorsal in supine position, ventral in prone position) lung regions in low PEEP conditions. Transdiaphragmatic pressure reduction (*vs.* low PEEP + supine) is shown in (**B**). Transdiaphragmatic pressure is reduced by higher PEEP (regardless of body position) and prone position (regardless of PEEP levels). * p < 0.01 *vs*. all conditions; + p < 0.05 *vs*. low PEEP conditions. Abbreviations: CT computed tomography; HU Hounsfield unit; PEEP positive end-expiratory pressure; SD standard deviation

Transdiaphragmatic pressure was greater as stimulation frequency was higher in all conditions (Fig. 2). Overall, transdiaphragmatic pressure was the highest at low PEEP in supine position and the lowest at high PEEP in prone position (Fig. 2). Compared to values in low PEEP + supine, transdiaphragmatic pressure was significantly reduced by either changing position to prone alone (Low PEEP, Prone: -27.9 ± 30.9%) or increasing PEEP alone (High PEEP, Supine: -51.7 ± 16.6%) or both combinations (High PEEP, Prone: -62.0 ± 14.6%) (Fig. 3B).

CT analysis found that high PEEP in both positions decreased the amount of non-aeration (Table) and thus end-expiratory lung volume was significantly increased with increasing PEEP in both positions (Fig. 3A). Regardless of PEEP levels, however, end-expiratory lung volume was not altered by body position (Fig. 3A).

## Discussion

The current study found that in a rabbit ARDS model, the capacity of force generation from diaphragm was altered by PEEP and body position- transdiaphragmatic pressure following bilateral phrenic nerve stimulation was decreased by increasing PEEP (regardless of body position) and changing position from supine to prone (regardless of PEEP levels).

Two potential strategies were revealed to modulate the force generated by diaphragmatic contraction and thereby to mitigate effort-dependent lung injury in ARDS. *First*, higher PEEP was associated with less spontaneous breathing effort (reflected by esophageal pressure) during mechanical ventilation in ARDS[1, 11, 12, 18] although its distinct mechanism has been unknown. Here, we confirmed that PEEP was an independent mechanistic determinant of force generation from diaphragm by altering end-expiratory lung volume (evidenced by CT). A recent clinical study using healthy volunteer also showed higher PEEP decreased neuromechanical efficiency of the diaphragm [19]. Of course, higher lung volume is known to shorten diaphragm length, resulting in less force generation from diaphragm[9, 10].

*Second*, body position per se modulated the capacity of force generation from diaphragm. Transdiaphragmatic pressure was increased in supine position and decreased in prone position. This effect was independent of PEEP levels or end-expiratory lung volume. This is probably because prone position per se shorten diaphragm length even with the same end-expiratory lung volume due to altered chest wall configuration and diaphragm geometry[20, 21]. Our findings partially explain recent clinical observations. Prone position was associated with less spontaneous effort (estimated by esophageal pressure), resulting in less systemic inflammation in severe ARDS[13]. Further, awake prone position decreased respiratory rate and decreased the need of intubation[22]. Thus, the current study may reveal a protective mechanism of prone position to mitigate effort-dependent lung injury in ARDS.

The model studied here is well known to be a model of ‘recruitable’ lung and rabbits are naturally prone. Lung recruitment was calculated as the proportion of the total lung weight accounted for by nonaerated lung tissue (expressed in HU between + 100 and -200) in which aeration was restored by PEEP of 10 cmH_2_O and 3 cmH_2_O in supine position[23]. Indeed, lung recruitment calculated between PEEP of 3 and 10 cmH_2_O was 23 ± 15%, confirming that our surfactant-depleted rabbit ARDS model was very recruitable. In contrast, human ARDS usually has heterogeneous etiology, variable lung recruitability and the far longer usual time-course. Although the model studied here has been successfully used to illustrate key mechanism of the capacity of force generation from diaphragm, caution is necessary in extrapolating the current data to the clinical context. Further study would be necessary to investigate the impacts of PEEP and body position on the capacity of force generation from diaphragm in a model of ‘non-recruitable’ lung. In this study, prone position had no impact on end-expiratory lung volume. Previous review reported the impacts of prone position on end-expiratory lung volume were conflicting (probably depending on lung recruitablity, shape of chest wall, the presence of abdominal hypertension and the presence of support)[24].The long-term effect of strategy to alter the capacity of force generation from diaphragm is unknown and of note, a previous animal study showed that prolonged application of higher PEEP caused longitudinal muscle fiber atrophy[25].

## Conclusions

The capacity of force generation from diaphragm was modulated by PEEP and body position during mechanical ventilation in ARDS. Higher PEEP or prone position per se or both was effective to decrease the force generation from diaphragm.

## Data Availability

The datasets used and/or analyzed during the current study are available from the corresponding author on reasonable request.

## Abbreviations

ARDS: Acute Respiratory Distress Syndrome
PEEP: Positive End-Expiratory Pressure
CT: Computed Tomography
